# Epigenetic suppression of EGFR signaling in G-CIMP+ glioblastomas

**DOI:** 10.18632/oncotarget.2350

**Published:** 2014-08-16

**Authors:** Jie Li, Zachary J. Taich, Amit Goyal, David Gonda, Johnny Akers, Bandita Adhikari, Kunal Patel, Scott Vandenberg, Wei Yan, Zhaoshi Bao, Bob S. Carter, Renzhi Wang, Ying Mao, Tao Jiang, Clark C. Chen

**Affiliations:** ^1^ Center for Theoretical and Applied Neuro-Oncology, Division of Neurosurgery, University of California, San Diego; ^2^ Department of Pathology, University of California, San Diego; ^3^ Department of Neurosurgery, Tiantan Hospital, Capital Medical University, Beijing, China; ^4^ Department of Neurosurgery, Peking Union Medical College Hospital, Beijing, China; ^5^ Department of Neurosurgery, Huashan Hospital, Shanghai Medical College, Fudan University, Shanghai, China

**Keywords:** G-CIMP, EGFR, Glioblastoma, Epigenetic suppression

## Abstract

The intrinsic signaling cascades and cell states associated with the Glioma CpG Island Methylator Phenotype (G-CIMP) remain poorly understood. Using published mRNA signatures associated with EGFR activation, we demonstrate that G-CIMP+ tumors harbor decreased EGFR signaling using three independent datasets, including the Chinese Glioma Genome Atlas(CGGA; n=155), the REMBRANDT dataset (n=288), and The Cancer Genome Atlas (TCGA; n=406). Additionally, an independent collection of 25 fresh-frozen glioblastomas confirmed lowered pERK levels in G-CIMP+ specimens (p<0.001), indicating suppressed EGFR signaling. Analysis of TCGA glioblastomas revealed that G-CIMP+ glioblastomas harbored lowered mRNA levels for EGFR and H-Ras. Induction of G-CIMP+ state by exogenous expression of a mutated isocitrate dehydrogenase 1, *IDH1-R132H*, suppressed EGFR and H-Ras protein expression as well as pERK accumulation in independent glioblastoma models. These suppressions were associated with increased deposition of the repressive histone markers, H3K9me3 and H3K27me3, in the EGFR and H-Ras promoter regions. The *IDH1-R132H* expression-induced pERK suppression can be reversed by exogenous expression of H-RasG12V. Finally, the G-CIMP+ *Ink4a-Arf^−/−^ EGFRvIII* glioblastoma line was more resistant to the EGFR inhibitor, Gefitinib, relative to its isogenic G-CIMP- counterpart. These results suggest that G-CIMP epigenetically regulates EGFR signaling and serves as a predictive biomarker for EGFR inhibitors in glioblastoma patients.

## INTRODUCTION

Glioblastoma is the most common form of primary brain cancer and remains one of the most devastating of human diseases [1]. The aggregate of laboratory and clinical investigations spanning the past four decades has led to the understanding that glioblastomas, like most cancers, are defined by a unifying set of phenotypes, including self-sufficiency in growth signaling and altered DNA damage response [2, 3]. However, the underlying molecular events responsible for these phenotypes are diverse, and they vary among different glioblastomas. Thus, the term glioblastoma captures a wide spectrum of molecular physiologies [4]. Meaningful therapeutic efforts will only be possible with the elucidation of these distinct physiologies [5].

One of the recurrent physiologic states in glioblastoma is the Glioma CpG island methylator phenotype (G-CIMP) [6]. Glioblastoma with this phenotype harbors extensive methylation in the CpG islands of a large number of genetic loci. The methylation pattern results from aberrant production of 2-hydroxyglutarate (2HG) by mutated forms of the isocitrate dehydrogenase genes (*IDH1* or *IDH2*) [7]. Nearly all IDH mutations associated with G-CIMP involve substitution of arginine 132 of *IDH1* with histidine (*IDH1-R132H*). The aberrant methylation induced by IDH mutations, in turn, down-regulates expression of select genes, resulting in a unique physiologic state [6]. As such, it is not surprising that the epidemiology and clinical course of G-CIMP+ glioblastomas differ significantly from those of G-CIMP- glioblastomas [6]. While it is clear that G-CIMP+ glioblastomas harbor a distinct biology, the intrinsic cellular physiology contributing to this biology remains poorly understood.

The intrinsic physiology of a cell state is largely driven by intracellular signal transduction cascades [8]. For glioblastomas, receptor tyrosine kinase (RTK) signaling plays critical roles in tumor initiation and maintenance [9]. The Epidermal Growth Factor Receptor (EGFR), in particular, is a RTK that is mutated, amplified, or hyperactive in nearly all glioblastomas [9]. We wished to determine whether G-CIMP+ and G-CIMP- glioblastomas differentially utilize these signaling cascades. *In vitro* studies suggest that signal pathway activation triggers physiologic changes that can be reliably measured by altered mRNA expression [10]. In our study, we utilized these mRNA signatures as a platform for analyzing transcriptome datasets derived from clinical glioblastoma specimens. Using this platform, we showed the EGFR signaling was suppressed in G-CIMP+ glioblastomas. Moreover, our results suggest that induction of the G-CIMP+ state is associated with suppression of EGFR and H-Ras expression, resulting in suppressed EGFR signaling.

## RESULTS

### Identification of gene signatures

**Figure 1:**
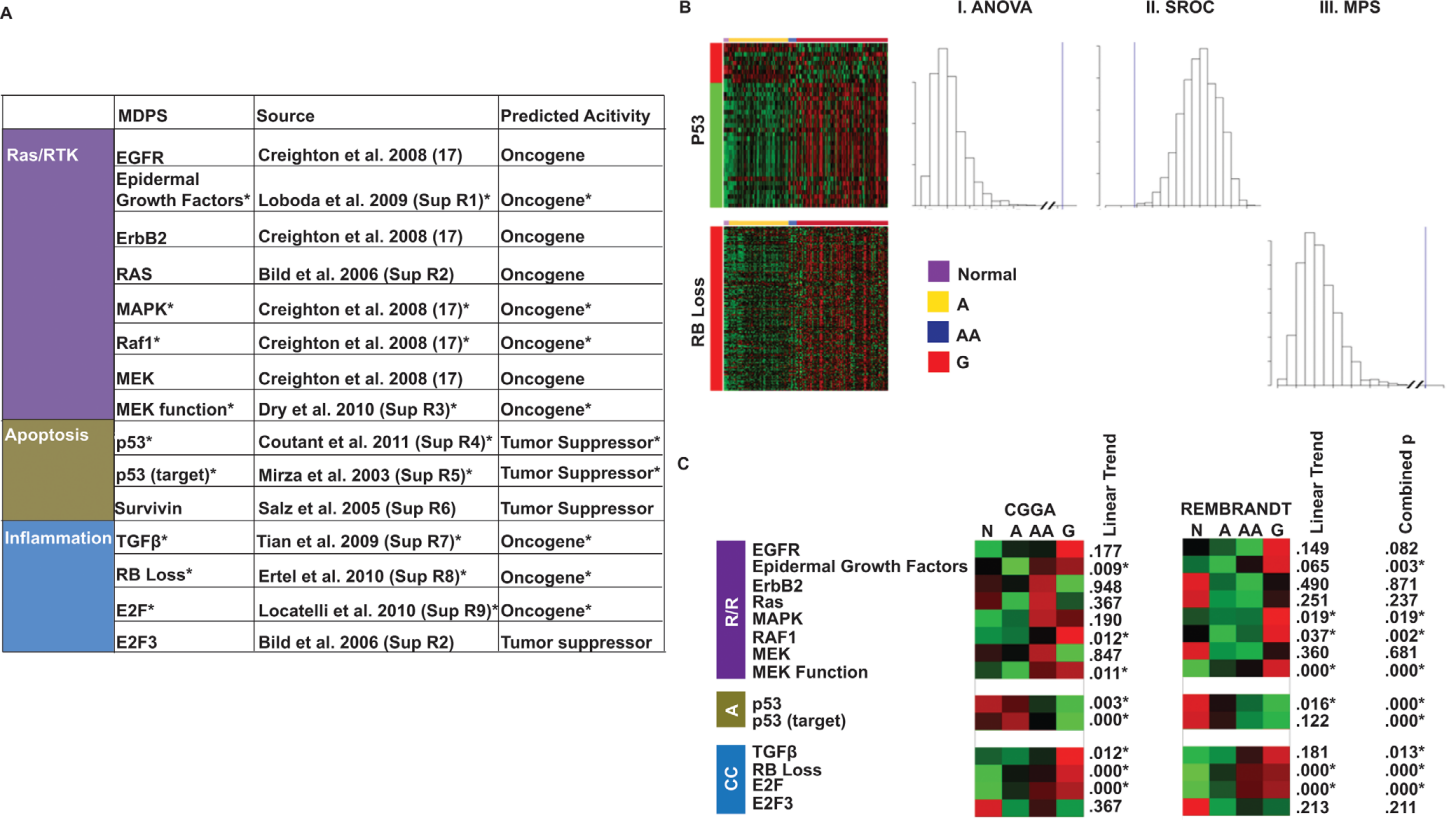
Identification and validation of gene signatures (A) Published gene signatures that captured the activation of canonical signaling pathways as described by Hanahan and Weinberg [2]. Indicated with * are the signatures that were validated by the internal consistency and the biologic plausibility test (see Methods). (B) Test of internal consistency. The heat map shows the expression of the p53 signature genes in the CGGA data set. The gene annotations on the left side show which genes are parts of the up- (red) and down- (green) regulated components of the signature. Distribution of the ANOVA and SROC statistics were empirically derived for each signature by a bootstrapping procedure (see Methods) in which 1500 Monte-Carlo simulations were performed. For signatures consisting of only over- or under-expressed genes (e.g. RB Loss), the mean pair-wise SROC between all genes in the signature was calculated and simulated. The blue line indicates where the actual expression of signature genes in the clinical specimen falls within this distribution. (C) Test of biologic consistency. Collapsed gene signature heat maps showing the mean expression of the gene signature in normal (N), grade II glioma (a.k.a. astrocytoma, “A”), grade III glioma (a.k.a. anaplastic astrocytoma, “AA”), and grade IV glioma (a.k.a. glioblastoma, “G”) in both the CGGA and REMBRANDT data set. The linear trend p is the bootstrapped one-tailed p from 1500 simulations of the Kendall Tau rank correlation coefficient. The combined p statistic is from the Stouffer Weighted combination of the p values from each data set for each gene signature. Signatures with combined p values < .05 were included in later analyses.

The TCGA efforts have identified three pathways that are aberrantly regulated in glioblastomas, including those mediated by RTKs, p53, and Rb. We performed an exhaustive search of the literature to identify mRNA signatures that captured the activation of these pathways (Figure 1A). Gene signatures reflecting RTK pathway activity include: PTEN loss, EGFR, ErbB2, Ras, MAPK, RAF1, MEK, MEK Function, and Src. Gene signatures that captured Rb pathway activity include: Rb loss, E2F, and E2F3. Several gene signatures related to apoptosis and DNA damage response were identified, including p53, p53 target, and Survivin.

### Validation of internal consistency

We filtered these gene signatures through two validation steps. First, we reasoned that if the signature harbors biologic meaning in clinical glioblastoma specimens, then the general pattern of gene expression described by the signature should be grossly conserved in the mRNA profiles of clinical specimens. That is, genes that are up-regulated in the signatures should cluster in terms of their expression pattern in the clinical specimen. Moreover, these genes should more likely be over-expressed in clinical specimens than in a random set of genes. Analogous predictions are made for the genes that are under-expressed. We refer to this test as a validation for “internal consistency.” We tested this consistency using mRNA profiles derived from clinical glioma specimens in the REMBRANDT (n=288) and the CGGA (n=155) data sets using the ANOVA and SROC statistics (see Methods). Overall, 79% of the published signatures passed the internal consistency test in both datasets (Figure 1).

To understand the interplay between the gene signatures, we determined the extent of overlap between the various gene lists for each signaling pathway (Supplemental Figure 1). The highest overlaps are between MAPK and RAF1 where 62.98% of the genes in the MAPK signature are in the RAF1 signature. However, these signatures are defined by the same study [31] and may be prone to systematic biases. Fortunately, other signatures of Ras/RTK activation reported by independent groups were identified in our search (See Figure 1A). In contrast, the p53 signatures share only 0.4-1.6 % of the genes. The low level of overlap in most signatures suggests that these signatures offer relatively independent assessment of the pathway's activity.

### Validation of biologic relevance

The prevailing model of carcinogenesis suggests progressive or step-wise increase in oncogenic signaling and diminution of tumor suppressor signaling during tumor progression secondary to accumulation of genetic and epigenetic changes [2]. For instance, progressive increases in RTK signaling have been noted with advancing grades during glioma pathogenesis [3]. Similarly, abridgement of DNA damage response mediated by p53 is a critical step during transition from lower grade glioma to higher grade glioma [32]. For gene signatures to be biologically relevant, they should capture this biology.

To assess the expression patterns of our gene signatures as a function of glioma grade, we collapsed the gene signature for each specimen of the same tumor grade into a single value [33] (see Methods). This average pathway activity score was converted into a heat map for visual display, with “red” denoting increased pathway activity and “green” denoting decreased pathway activity (Supplemental Figure 2, Figure 1B and C). We then performed statistical analysis to identify signatures that i) trended with progressive tumor grades or ii) exhibited differing activity score between grade 2 and 4 gliomas. Through this analysis, we identified four signatures associated with RTK activation (Epidermal Growth Factor, MAPK, RAF1, and MEK), three signatures associated with Rb pathway inactivation (TGF-β, Rb loss and E2F), and three signatures associated with p53 pathway activation (p53 and p53 targets). Overall, 68% of the internally consistent gene signatures passed the biological plausibility test. The genes that make up of the various signatures can be found in Supplemental Table 2.

### Transcriptome based determination of G-CIMP status

Since the CGGA and the REMBRANDT glioblastoma specimens were not subjected to global genomic methylation profiling, the samples cannot be directly assessed for G-CIMP status. However, mRNA based gene classifiers for G-CIMP+ tumors have been reported [22]. We used the Prediction Algorithm for Microarrays (PAM) and these classifiers to categorize tumors as either G-CIMP+ or G-CIMP-. Since *IDH1* mutational status is available in the CGGA and *IDH1* mutation is tightly coupled to G-CIMP+ status [7], we used *IDH1* mutation as a proxy for G-CIMP positivity. Our PAM classified *IDH1* mutated gliomas with a sensitivity of 95.65% and specificity of 92.41%. We subsequently tested the method using the subset of glioblastomas in the TCGA where G-CIMP status was directly determined. In this analysis, PAM application of the G-CIMP signature discriminated G-CIMP status with 97.95% sensitivity and 85.67% specificity (Supplemental Figure 3). We therefore conclude that the method of G-CIMP discrimination by mRNA signature is robust, allowing us to interrogate the complete CGGA and REMBRANDT data sets.

### Differential pathway utilization in G-CIMP+ and G-CIMP- tumor

Using PAM application of G-CIMP mRNA signature, we categorized glioblastoma specimens in the CGGA and the REMBRANDT as either G-CIMP+ or G-CIMP-. We then compared the activity score of the various gene signatures in the G-CIMP+ and the G-CIMP- tumor.

**Figure 2:**
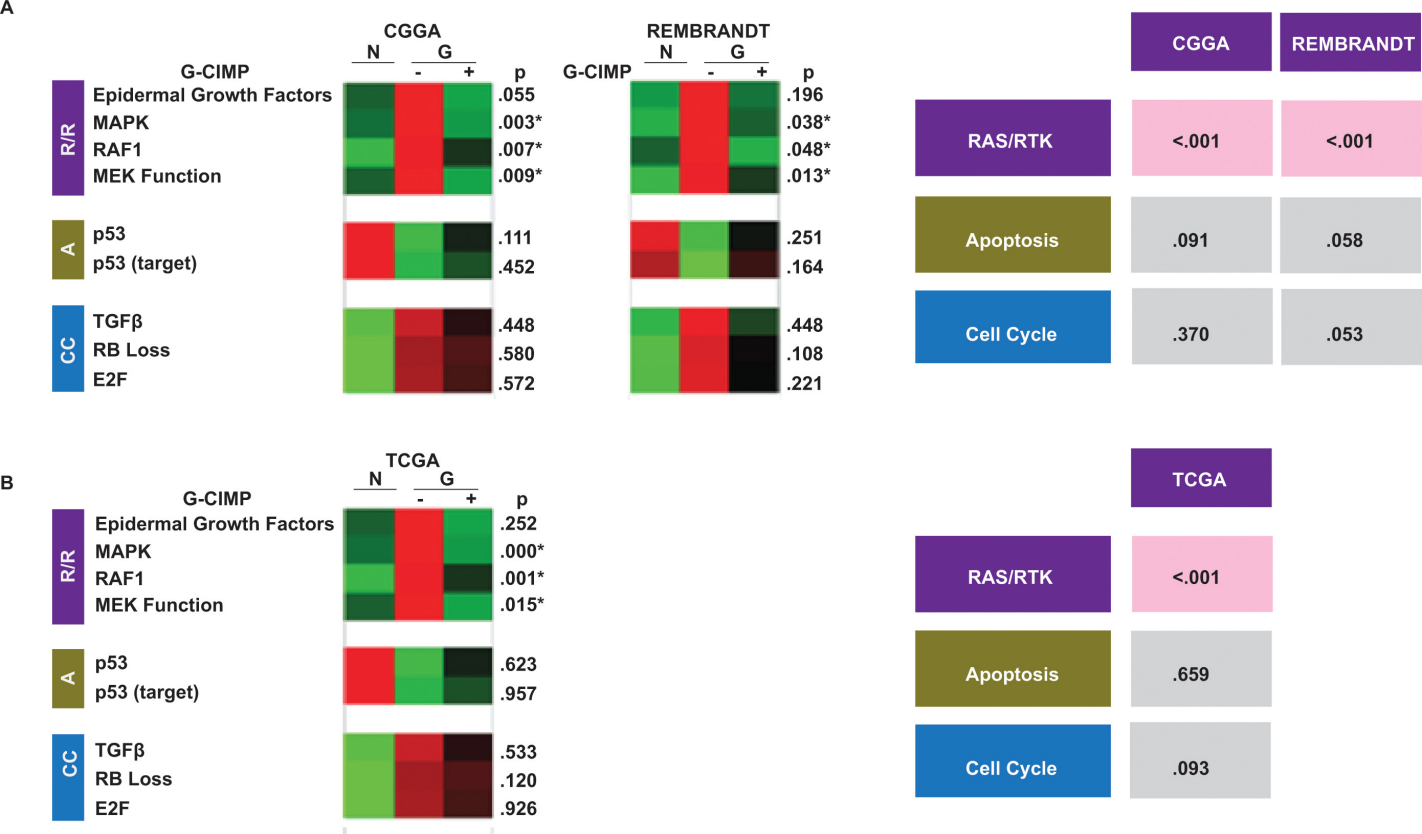
Differential pathway utilization in G-CIMP+ and G-CIMP- glioblastomas (A) Differing signature expression by G-CIMP status. Left: collapsed gene signature heat maps for the validated gene signatures in normal (N) and G-CIMP-, and G-CIMP+ glioblastomas (“G”) profiled in the CGGA and REMBRANDT data sets. p values are bootstrapped two-tailed t tests between G-CIMP+ and G-CIMP- glioblastomas. Right: Bootstrapped p-values for the combined two-tailed t tests for each signature group. p < 0.05 was boxed in red. G-CIMP status of samples in the CGGA and REMEBRANDT was determined based on PAM classifiers. (B) Validation in the methylation-profiled TCGA data set. Left: collapsed gene signature heat maps for the validated gene signatures in normal (N) and G-CIMP-, and G-CIMP+ glioblastomas (“G”) profiled in the TCGA. Right: Bootstrapped p-values for the combined two-tailed t tests for each signature group. p-value < 0.05 was boxed in red. G-CIMP status in the TCGA dataset is determined using global genomic methylation profiles as described by Noushmehr *et al*. [6].

The results of this analysis are remarkably consistent. For EGFR pathway activation, the activity scores for the EGFR, MAPK, RAF1, and MEK signatures were higher in the G-CIMP- glioblastomas in the CGGA. This difference appeared notable for the MAPK, RAF1 and MEK signatures (p=0.003, 0.007 and 0.009 respectively, see Methods). Moreover, the results were highly reproducible within the REMBRANDT data set. On the other hand, the pathway activity scores associated with Rb Loss and p53 did not differ based on G-CIMP status (Figure 2A, left panel).

As a means to derive definitive statistics for the overall status of the canonical pathways represented by multiple sub-signatures, the individual statistics for the contributing signatures were combined using the Stouffer Weighted Z score (see Methods). This analysis enrichment of the Ras/RTK (EGFR, MAPK, RAF1, and MEK) in G-CIMP- glioblastomas is relative to the G-CIMP+ glioblastomas in both the CGGA and the REMBRANDT dataset. On the other hand, Rb loss and p53 inactivation signatures did not differ based on G-CIMP status (Figure 2A, right panel).

### Validation of observation using the TCGA glioblastoma dataset

To validate our findings, we turned to the subset of glioblastomas in the TCGA data base (n=406) that were profiled for both global methylation status as well as overall mRNA expression. The glioblastomas were categorized by G-CIMP status based on direct methylation profiling. Overall pathway activity was determined as above-described. The TCGA results faithfully recapitulated those observed in the CGGA and REMBRANDT. Namely, RTK pathway score was consistently elevated in G-CIMP- glioblastomas relative to G-CIMP+ glioblastomas (p value of <.001). Pathway activity scores associated with Rb inactivation and p53 activation, as in the discovery data sets, did not significantly differ based on G-CIMP status (Figure 2B).

### Validation by pERK Western blotting

**Figure 3:**
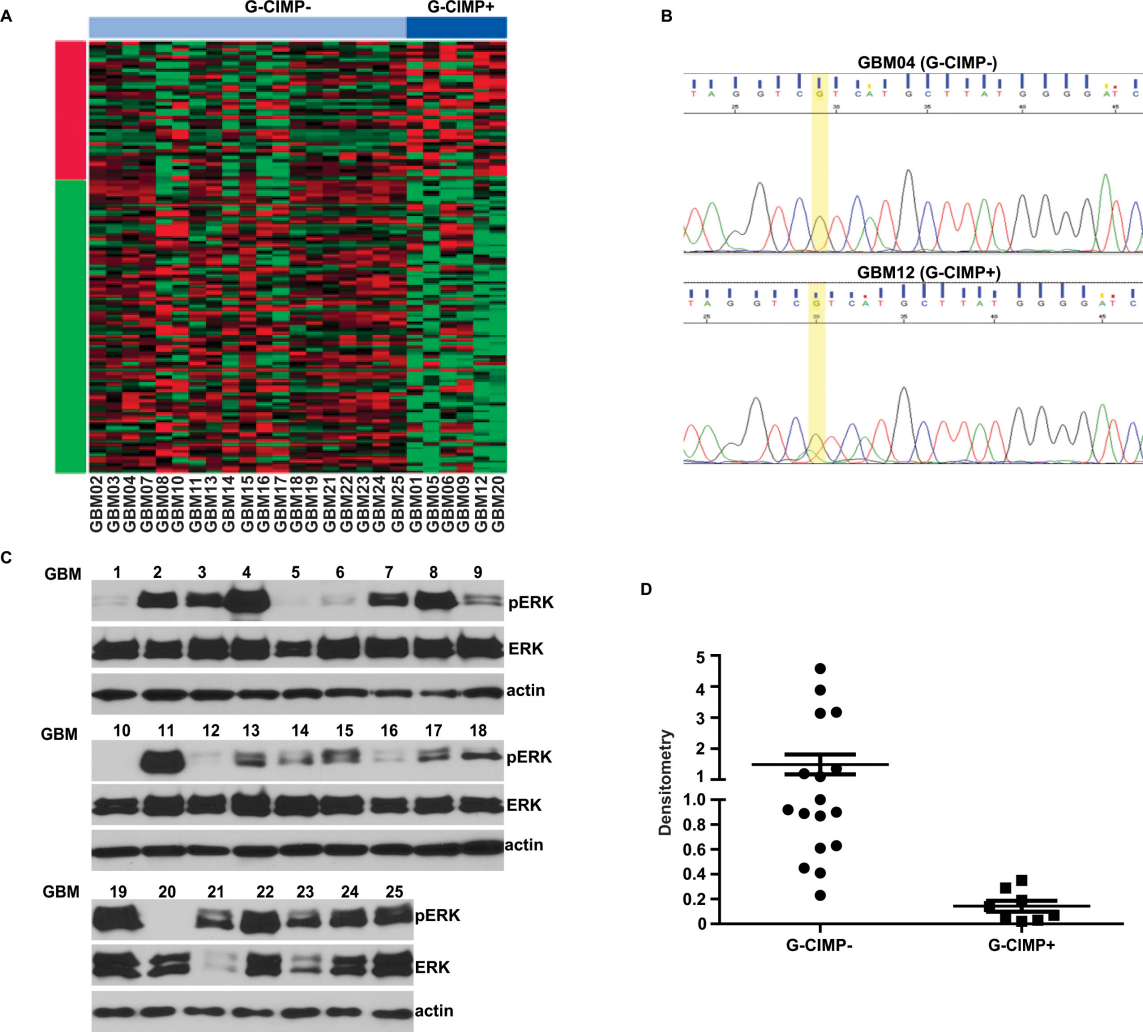
Decreased pERK signaling in G-CIMP+ glioblastomas (A) G-CIMP status of an independent collection of 25 glioblastoma specimens. These specimens were transcriptomally profiled and classified into G-CIMP+ and G-CIMP- using the PAM classifier described above. 6 G-CIMP+ and 19 G-CIMP- glioblastomas were identified. (B) Representative image of Chromatogram profile after direct DNA sequencing showing *IDH1* mutations in G-CIMP+ glioblastoma samples. All specimens predicted by PAM to be G-CIMP+ harbored the *IDH1-R132H* mutation. (C) Decreased pERK level in G-CIMP+ glioblastoma specimens. Ras/RTK signaling was analyzed by Western blotting using an antibody against pERK, an established biomarker for Ras/RTK pathway activity. (D) Levels of pERK, ERK and actin in (C) were analyzed using Image J software (Bethesda, MD). The level of pERK was normalized (see Methods) and plotted as a function of G-CIMP status.

To further verify that the results of our comparative gene signature analysis reflect genuine biology, we tested whether G-CIMP+ and G-CIMP- glioblastoma specimens harbor differing levels of pERK, a biomarker of EGFR pathway activation [34]. 25 additional glioblastoma specimens were transcriptomally profiled and classified into G-CIMP+ and G-CIMP- using the PAM classifier described above. 6 G-CIMP+ and 19 G-CIMP- glioblastomas were identified (Figure 3A). Consistent with prior reports, G-CIMP+ glioblastomas all harbored the *IDH1-R132H* mutation (Figure 3B). The level of pERK was significantly higher in the G-CIMP- specimens relative to the G-CIMP+ specimens (Figure 3C and D). These results support our gene signature analysis and suggest differential activation of the RTK/Ras pathway in G-CIMP+ and G-CIMP- glioblastomas.

### Suppression of EGFR expression in G-CIMP+ glioblastomas

**Figure 4:**
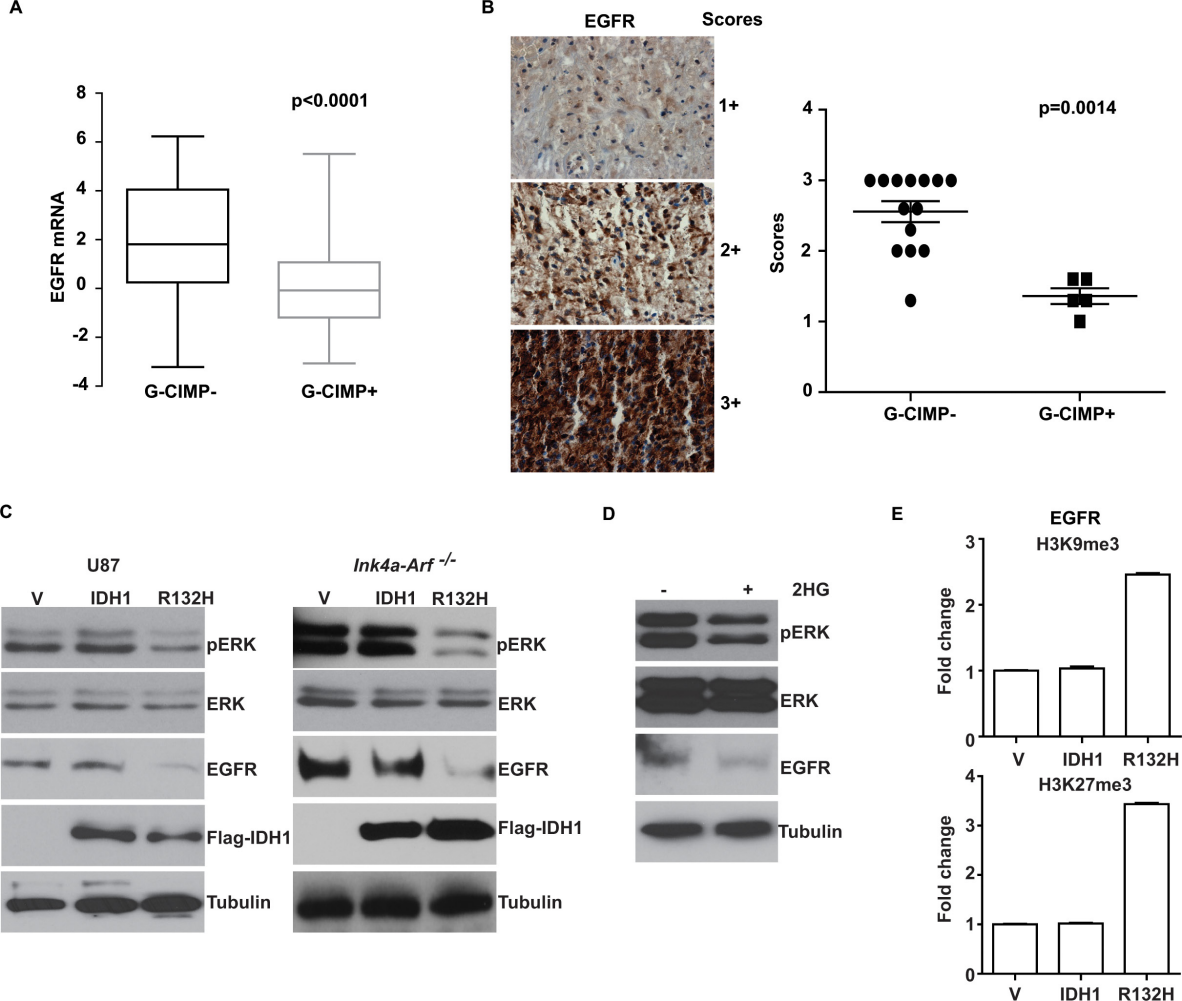
Suppression of EGFR expression in G-CIMP+ glioblastomas (A) Decreased EGFR mRNA level in G-CIMP+ TCGA glioblastomas. G-CIMP status in the TCGA dataset is determined using global genomic methylation profiles as described by Noushmehr *et al*. [6]. (B) IHC staining using TMA of 19 independent glioblastoma specimens (5 G-CIMP+ and 14 G-CIMP-; G-CIMP assignment made based on PAM classifiers). Left: Representative images of IHC staining and grading scheme. Right: Scoring of IHC staining was plotted as a function of G-CIMP status. p value was derived using the non-parametric Mann-Whitney test as described in Methods. (C) Induction of G-CIMP+ status suppressed EGFR expression and pERK accumulation. Human U87 MG glioblastoma cells or murine *Ink4a-Arf^−/−^* glioblastoma line were stably transduced with retrovirus carrying empty vector, *IDH1* or *IDH1-R132H*. Cells were propagated for >10 passages. Whole cell lysates were extracted and analyzed with Western blotting using antibodies against pERK, ERK, EGFR, Flag (for IDH1) and Tubulin (as loading control). (D) Induction of G-CIMP+ status by treatment of U87MG cell with 2HG suppressed EGFR expression and pERK accumulation. U87MG cells were treated with 2HG (1mM) and passaged for >10 generations. Whole cell lysates were then collected and analyzed by immunoblotting with antibodies against pERK, ERK, EGFR and Tubulin (as loading control). (E) Induction of G-CIMP+ state increased deposition of repressive histone markers H3K9me3 and H3K27me3 in the EGFR promoter region. U87MG cells stably transduced with retrovirus carrying empty vector, *IDH1* or *IDH1-R132H* mutant were lysed after crosslinking with formaldehyde. Chromatin was extracted, fragmented, and immunoprecipitated with antibodies against Histone H3, H3K9me3 and H3K27me3. Relative abundance of H3K9me3 and H3K27me3 at EGFR promoter region was shown as fold change compared to the cells transduced with empty vector. Error bars represent standard deviation.

Subtle differences in EGFR expression level are amplified by cascade interactions into significant differences in signaling output [35]. Thus, we tested the hypothesis that the lowered EGFR signaling in G-CIMP+ glioblastomas was related to lowered EGFR expression in these tumors. To this end, we used the TCGA glioblastoma data set, where tumors are classified by G-CIMP status based on global genomic methylation profiles. We found that G-CIMP+ tumors expressed lower levels of EGFR mRNAs relative to G-CIMP- tumors (Figure 4A). Additionally, we validated this observation by EGFR immunohistochemistry (IHC) using a tissue microarray (TMA) consisting of 19 independent glioblastoma specimens (5 G-CIMP+ and 14 G-CIMP-, p=0.0014, Figure 4B).

These results suggest that the induction of a G-CIMP+ state may suppress EGFR expression. To test this hypothesis, we exogenously expressed *IDH1-R132H* to induce a G-CIMP+ state [7] in the human U87MG glioblastoma line as well as an *Ink4a-Arf^−/−^* genetically engineered murine model (GEMM) derived glioblastoma line [36]. In both models, exogenous expression of *IDH1-R132H* was associated with suppression of EGFR expression (Figure 4C). Further supporting our hypothesis, induction of G-CIMP+ status in U87MG by chronic treatment with 2HG [37] also induced a reduction in EGFR expression (Figure 4D).

The prevailing model of the biology of G-CIMP+ tumors involves epigenetic regulation of gene expression [38]. As such, we wished to determine whether the G-CIMP+ related EGFR suppression was related to deposition of repressive histone markers [39] in the region of the EGFR promoter. To this end, ChIP analysis was performed to determine whether *IDH1-R132H* expression was associated with the increased deposition of H3K9me3 and H3K27me3, two well-established repressive histone markers that are associated with transcriptional repression [40], in the EGFR promoter region. In the U87MG model, exogenous expression of *IDH1-R132H* was associated with increased deposition of H3K9me3 and H3K27me3 in the EGFR promoter region, suggesting that G-CIMP+ state is epigenetic down-regulation of EGFR (Figure 4E).

### Suppression of H-Ras expression in G-CIMP+ glioblastomas

**Figure 5:**
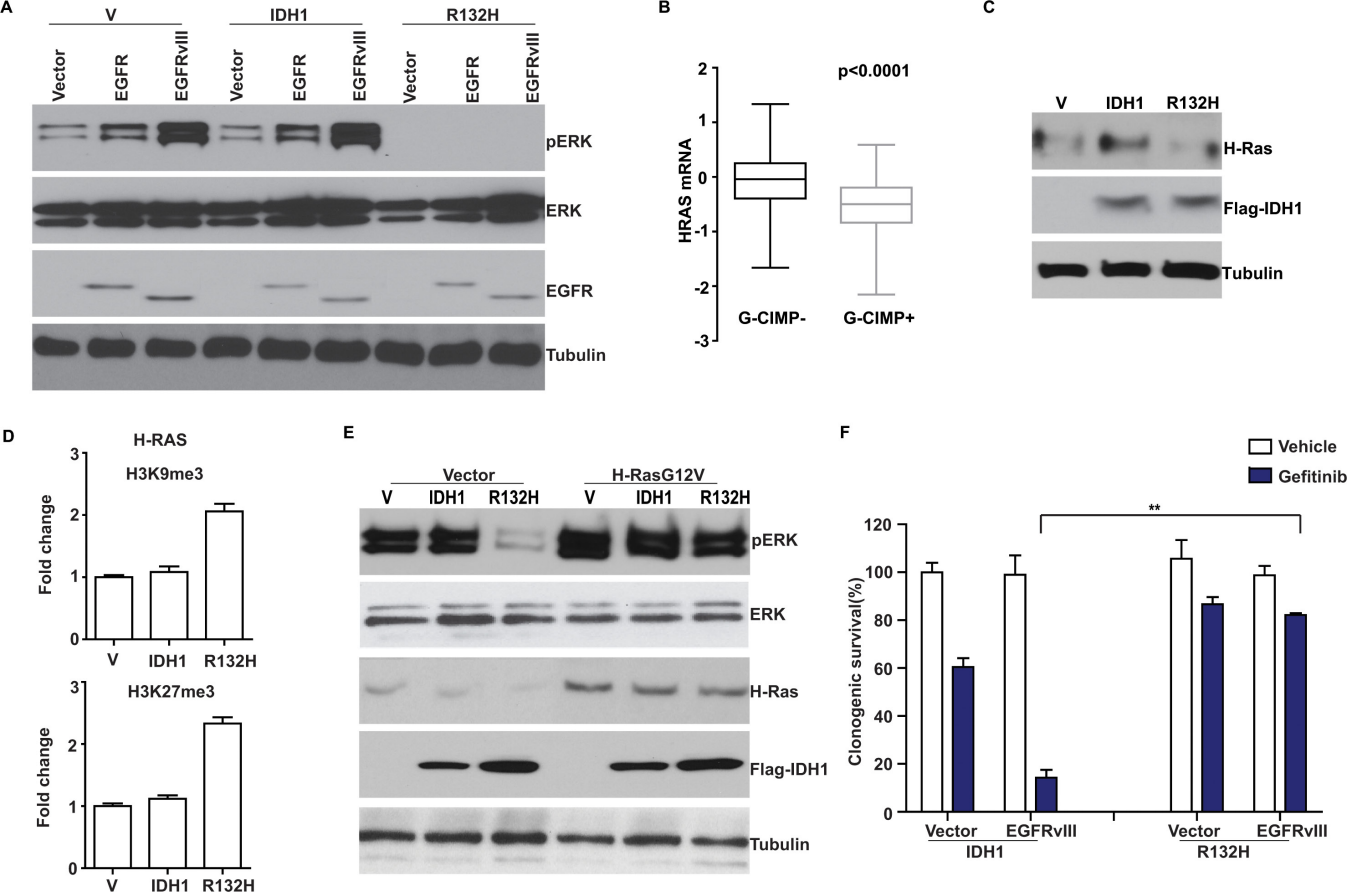
Suppression of H-Ras expression in G-CIMP+ glioblastomas (A) Ectopic expression of EGFR or EGFRvIII failed to restore pERK accumulation in G-CIMP+ *Ink4a-Arf^−/−^* glioblastoma cells. *Ink4a-Arf^−/−^* glioblastoma lines expressing wild type or mutated (*R132H*) *IDH1* were stably transduced with retrovirus carrying vector, EGFR or EGFRvIII expression construct, separately. Whole cell lysates were extracted and analyzed with Western blotting using antibodies against pERK, ERK, EGFR and Tubulin (as loading control). (B) Decreased H-Ras mRNA level in G-CIMP+ TCGA glioblastomas. G-CIMP status in the TCGA dataset is determined using global genomic methylation profiles as described by Noushmehr *et al*. [6]. (C) Induction of G-CIMP+ status suppressed H-Ras expression. Human U87 MG glioblastoma line was stably transduced with retrovirus carrying empty vector, *IDH1* or *IDH1-R132H*. Cells were propagated for >10 passages. Whole cell lysates were extracted and analyzed with Western blotting using antibodies against H-Ras, Flag (for IDH1), and Tubulin (as loading control). (D) Induction of G-CIMP+ state increased deposition of repressive histone markers H3K9me3 and H3K27me3 in the H-Ras promoter region. U87 cells stably transduced with retrovirus carrying empty vector, *IDH1* or *IDH1-R132H* mutant were lysed after crosslinking with formaldehyde. Chromatin was extracted, fragmented, and immunoprecipitated with antibodies against Histone H3, H3K9me3 and H3K27me3. Relative abundance of H3K9me3 and H3K27me3 at H-Ras promoter region was shown as fold change compared to the cells transduced with empty vector. Error bars represent standard deviation. (E) Ectopic expression of H-Ras restored pERK accumulation in G-CIMP+ *Ink4a-Arf^−/−^ EGFRvIII* glioblastoma cells. H-Ras expression constructs were stably transfected into *Ink4a-Arf^−/−^* glioblastoma lines expressing wild type or mutated (*R132H*) *IDH1*. Whole cell lysates were extracted and analyzed with Western blotting using antibodies against pERK, ERK, H-Ras, Flag (for IDH1), and Tubulin (as loading control). (F) G-CIMP+ state lowered glioblastoma sensitivity to the EGFR inhibitor, Gefitinib. Murine *Ink4a-Arf^−/−^* cells expressing wild type *IDH1* or *IDH1-R132H* were stably transduced with empty vector or *EGFRvIII* and treated with vehicle or Gefitinib at 10 μM for 14 days. Clonogenic survival was determined. ** indicates statistical significance at p<0.01.

If suppression of EGFR expression is the sole mechanism by which G-CIMP+ glioblastomas down-regulate EGFR signaling, then exogenous expression of EGFR should restore this signaling. We tested this hypothesis. Surprisingly, exogenous expression of neither a wild type EGFR nor a hyperactive, oncogenic form of EGFR, EGFRvIII restored pERK accumulation in the G-CIMP+ *Ink4-Arf^−/−^*glioblastoma line (Figure 5A). This result suggests that G-CIMP+ status may additionally suppress the expression of genes down-stream of EGFR that are required for EGFR signaling.

To identify such genes, we tested whether downstream effectors of EGFR, including H-Ras, were differentially expressed based on G-CIMP status. Using the TCGA dataset, we found that H-Ras mRNA levels were significantly lowered in G-CIMP+ glioblastomas relative to G-CIMP- glioblastomas (Figure 5B). Moreover, exogenous expression of *IDH1-R132H* was associated with suppression of H-Ras expression in the U87MG model (Figure 5C). As was observed with EGFR, exogenous expression of *IDH1-R132H* was associated with increased deposition of two repressive histone markers, H3K9me3 and H3K27me3, in the promoter region of H-Ras (Figure 5D).

These results suggest that the lowered expression of H-Ras is a rate-limiting step for EGFR signaling in G-CIMP+ glioblastomas. To test this hypothesis, we tested how exogenous expression of a hyperactive form of H-Ras (H-RasG12V) restored pERK accumulation in the G-CIMP+ *Ink4a-Arf^−/−^ EGFRvIII* model. Supporting our hypothesis, exogenous H-RasG12V expression restored pERK accumulation to levels comparable to those of *Ink4a-Arf^−/−^ EGFRvIII* lines expressing wild-type IDH1 (Figure 5E).

The down-regulation of EGFR signaling suggests that G-CIMP+ glioblastomas may be less dependent on this mitogenic signaling pathway. As such, these tumors may be less sensitive to EGFR inhibitors relative to G-CIMP- glioblastomas. To test this hypothesis, we treated isogenic pairs of murine *Ink4a-Arf^−/−^ EGFRvIII* glioblastoma lines expressing either *IDH1-R132H* or *wild type IDH1* with the EGFR inhibitor, Gefitinib [41]. We found that the *Ink4a-Arf^−/−^ EGFRvIII* expressing the wild type *IDH1*exhibited exquisite Gefitinib sensitivity (Figure 5F). In contrast, the *Ink4a-Arf^−/−^ EGFRvIII IDH1-R132H* cells exhibited a near 10-fold increase in Gefitinib resistance [36]. These results suggest that G-CIMP status of glioblastomas influences cellular sensitivity to EGFR inhibitors.

## DISCUSSION

With the advent of high-throughput genomic technologies including transcriptomal profiling and the application of these platforms to clinical tumor specimens, it is now possible to directly assess *in vivo* tumor physiology [42]. However, the biologic interpretations of these results are often difficult without insights derived from *in vitro* models. A major barrier in our ability to maximally extrapolate the clinical genomic information involves the lack of a reliable platform that affords translation of genomic information into biologic insight. It is, in this context, that the findings in this study are important. We used multiple methods to assess the internal consistency and biologic relevance of gene signatures derived using tissue culture models as they pertain to clinical specimens. We imposed strict criteria for these validation methods and demonstrate that signatures derived from tissue culture can be applied to clinical transcriptomal datasets to attain biologic insights. Importantly, signatures developed to reflect similar biology (e.g. EGFR activation) exhibit highly predictable and reproducible patterns of expression. Moreover, the results of our comparative pathway signature analysis are highly robust to the distinct profiling platforms utilized by different consortiums [43] as well as validation through cell biologic, proteomic [44], and immunohistochemical analysis.

Our results indicate that canonical pathways in glioblastoma biology can be generally divided into those that define the cancer phenotype and those that define molecular glioblastoma subtypes (such as G-CIMP positivity). Pathways regulating DNA damage response and cell cycle progression fall into the former category. Dysregulation of these processes is required early during carcinogenesis and likely occurs prior to genetic events that subsequently define molecular subtype [45]. For instance, inactivation of the p53 axis (either through mutations in genes required for p53 function or epigenetic modulation of these genes) is required to abridge cell cycle arrest that occurs in response to oncogenic events [46]. Disruption of this anti-tumor barrier is required early during carcinogenesis and is required for all cellular transformations [32, 47]. On the other hand, our current biologic understanding of glioblastoma pathogenesis suggests that EGFR activation occurs later during the evolution of the cancer [48]. The finding that these axes were hypoactive in G-CIMP+ glioblastomas suggests that the global methylation pattern or aspects of IDH biology prevents genetic/epigenetic events required for activation of these pathways or obviate the need for these pathways by functionally redundant circuitry.

Our analysis further highlights the complementary nature of comparative pathway signature analysis to genomic mutational analysis. For instance, G-CIMP+ glioblastomas are more likely associated with p53 mutations [4]. However, our comparative pathway signature analysis demonstrated that the p53 axis is inactivated early during glioblastoma pathogenesis in both G-CIMP+ and G-CIMP- tumor. Notably, the p53 axis can be inactivated by a multitude of mechanisms, including MDM2 amplification [49]. In this context, our data suggest that the mechanism of p53 axis inactivation differs between G-CIMP+ and G-CIMP- glioblastomas.

The finding that G-CIMP+ glioblastomas harbor lowered levels of EGFR signaling is largely consistent with the previous observation that IDH mutated, G-CIMP+ glioblastomas exhibited lowered likelihood of PTEN loss of heterozygosity or EGFR amplification [4]. Additionally, here we demonstrate that promoter methylation patterns in G-CIMP+ glioblastoma facilitated deposition of repressive histone markers in the promoter region of EGFR and H-Ras, thereby suppressing expression of these genes. This suppression, in turn, contributed to lower EGFR signaling in G-CIMP+ tumors. Decreased EGFR signaling in the G-CIMP+ glioblastomas suggest that these pathways may be peripheral in sustaining tumor growth and viability [50]. Supporting this hypothesis, we found that G-CIMP+ *Ink4a-Arf^−/−^ EGFRvIII* glioblastomas were more resistant to EGFR inhibition than their isogenic G-CIMP- counterparts. As such, consideration should be given to exclude G-CIMP+ glioblastoma patients from EGFR or RTK related therapeutic trials.

In conclusion, our study indicates that comparative pathway signature analyses using *in vitro* derived transcriptional signatures offer a robust methodology for understanding of glioblastoma physiology *in vivo*. Using this analytic approach, we found that EGFR signaling is suppressed in G-CIMP+ glioblastomas through epigenetic regulation. Importantly, our results were validated in four independent glioblastoma cohorts, totaling over 900 patients, with specimens profiled using distinct platforms. The consistency of the results observed in these independent datasets is both impressive and reassuring. These results harbor important clinical implications for therapeutic strategies targeting G-CIMP status or RTK signaling.

## MATERIALS AND METHODS

### mRNA microarray data

Exploratory studies were performed using the Chinese Glioma Genome Atlas (CGGA) and the Repository of Molecular Brain Neoplasia Data (REMBRANDT) datasets. The CGGA data set was kindly provided by Dr. Tao Jiang as normalized, probe-level expression values. Values of probes designed to assess the same gene were averaged [11]. REMBRANDT samples were downloaded from the caArray archive (https://array.nci.nih.gov/caarray/project/fine-00037) on March 13^th^, 2014 as raw, un-normalized CEL files. A Robust Multi-array Average (RMA) procedure was performed with a gene-based probe set for the HG-U133-Plus2 platform using the R-based *aroma.affymetrix* package [11].

Validation studies were performed using data from the Cancer Genome Atlas (TCGA). TCGA data was downloaded from the TCGA data portal (https://tcga-data.nci.nih.gov/tcga/) as level 3 data. Both mRNA expression and methylation profile microarray data for glioblastoma and normal brain specimens were downloaded. Descriptive statistics for the dataset are shown in Supplemental Table 1.

### Selection of gene signatures

The Frequency Weighted Links (FLink) tool was used to perform a comprehensive search for relevant gene signatures identified. Search terms, intended to identify multiple signatures in canonical signal transduction cascades, include: EGFR, Ras axis, Receptor Tyrsoine Kinase (RTK) activity. In addition to RTK signaling, we also identified signatures of pathways frequently altered in glioblastoma, including those mediated by Rb or p53 [12]. The signatures curated for this study are shown in Figure 1A.

### Characterizing the expression pattern of the gene signatures in clinical glioblastoma specimens

For a gene signature to be pertinent in clinical specimens, the expression patterns described by the signature should be conserved in the clinical specimens. That is, genes that are up-regulated in the signature should cluster in terms of their expression pattern in the clinical specimen. Moreover, these genes should be more likely over-expressed in clinical specimens than a randomly selected set of genes. Analogous predictions were made for the down-regulated genes in the signatures.

To test the conservation of gene expression patterns in a statistically rigorous manner, we took the following approach. First, if the expression pattern described by the gene signature is conserved in the clinical specimens, the variance (or variation) of the expression of the signature genes in the specimens should be largely explained by whether they were over- or under-expressed in the signature. To test this, a mean was calculated for the expression level of each signature gene across all samples. This mean was set to zero and the variance was set to one. We then utilized the F-statistics from ANOVAs to rigorously quantitate the variance of gene expression.

In performing these calculations, we avoided any assumptions that the F-statistics would follow any particular distribution. Instead, the distributions of these statistics were empirically derived for each signature by a bootstrapping procedure [13], in which 1500 Monte-Carlo simulations were performed where the genes within the signatures were randomized. For ANOVA, the p-value was 1 minus the percentile rank of the statistic within the bootstrapped distribution. A p-value was assigned to each gene signature. As an exploratory analysis, bootstrapped p-value of <0.05 was considered to be sufficient for further evaluation (Figure 1B). In general, we found this method to be more rigorous than parametric assumptions and multi-comparison correction [14].

Second, if the described signature expression pattern is conserved in clinical specimens, then these average expression values of the over- and under-expressed genes in the signature should be anti-correlated in the clinical specimen. This correlative analysis was done using the Spearman Rank Order Correlation (SROC). To determine the likelihood that any particular correlation coefficient would arise by pure chance, we performed 1500 Monte-Carlo simulations [15] where the genes within the signatures were randomized and SROC determined. For this correlative analysis, p-value is simply the percentile rank of the statistic within the Monte-Carlo distribution. A p-value was assigned to each signature. A p-value of <0.05 was considered to be sufficient for further evaluation (Figure 1B).

For signatures with only over-expressed genes, a Spearman Rank Order Correlation (SROC) was evaluated between all gene-pairs in the signature in terms of expression in clinical specimens. The mean of these values, termed “mean pair-wise spearman” (MPS) [16], was used to assess the extent to which the genes in the signatures are coordinately expressed in the clinical specimen. 1,500 Monte-Carlo simulations were then performed where the signature genes were randomized and MPS determined. The likelihood that the observed MPS for a particular signature will occur by pure chance was then determined from this distribution. A p-value of <0.05 was considered to be sufficient for further evaluation (Figure 1B).

Gene signatures harboring a p-value of <0.05 for the ANOVA and the SROC test or a p-value of <0.05 for the MPS analysis were selected for testing of biologic plausibility (see below).

### Defining pathway signature activity score

For each specimen, a pathway activity score was calculated using the t-score method developed by Creighton et al. [17]and used extensively in the TCGA studies [18]. In brief, each gene in the signature that was over-expressed was assigned the value of +1; genes that were under-expressed were assigned -1. The normalized expression values of the signature genes in the clinical specimen are then plotted against these assigned values, and Pearson Correlation Coefficient was determined. The correlation may range from -1 (denoting low pathway activity) to +1 (denoting high pathway activity). A schema of the method is shown in Supplemental Figure 2.

Several published signatures consist of only over-expressed genes. These signatures cannot be analyzed using the above described methods. Once again following the established method of Creighton et al.[17], for these “unidirectional” signatures, the pathway activity score is defined as the summed-average of the normalized expression value of the signature genes.

For ease of display, heat maps were generated to display the pathway activity scores for grade II, III, and IV gliomas as well as G-CIMP+ and G-CIMP- glioblastomas. For these heat maps, all pathway activity scores for each sample within a group (e.g. grade II gliomas) were collapsed into a mean value and displayed using a red (high expression)/green (low expression) color scheme (Supplemental Figure 2).

### Testing the biologic plausibility of the gene signatures

Advancing glioma grade is associated with progressively increasing oncogenic signaling and inactivation of tumor suppressor genes [1, 9]. In order for the gene signature to be biologically plausible, the expression of signature genes in the clinical specimens should recapitulate this expectation. Biological plausibility was evaluated for each of the internally consistent signatures in the CGGA and REMBRANDT data sets. The CGGA and REMBRANDT datasets were selected for analysis because both sets include transcriptome data for grade II, III, and IV gliomas. Pathway activity scores for all tumor samples were obtained using the methods described above. Changes in pathway activity score as a function of progressive tumor grades were assessed using a Kendall tau rank correlation coefficient test [19]. The test has the dual advantages of being based on rank, rather than serving as a test of linearity, and allowing for duplicated values, such as all tumors in a particular grade class being given the same tumor grade value.

The distribution of the Kendall Tau estimates for each signature in each data set was simulated 1500 times by Monte Carlo simulation [16]. As each signature had an *a priori* hypothesized trend direction, the percentile of the oncogenic signature in the distribution was used as the one-tailed p-value. The p-values from the analysis of the CGGA and the REMBRANDT data set were combined into a single p-value using the Stouffer Weighted Z method [20]. Signatures with a combined p-value less than .05 were considered to be validated for their biological plausibility (Figure 1C). Comparisons were additionally made between between grade II and IV gliomas using paired t-tests.

### Training G-CIMP Status

G-CIMP status was trained using the Prediction Analysis for Microarrays (PAM) algorithm, which classifies samples by nearest shrunken centroids [21]. In order to train the nearest shrunken centroid classifiers, *IDH1* mutation status data from the CGGA data was used as the gold standard of G-CIMP definition. *IDH1* mutated glioblastomas were defined as G-CIMP+ [1, 7], and *IDH1* wild-type glioblastomas were defined as G-CIMP-. PAM analysis was done using the 200 G-CIMP defining genes previously reported by Bayson *et al.* [22] as classifiers. A threshold was chosen to discriminate *IDH1* wild-type versus mutated tumors in the CGGA data set.

G-CIMP status in the TCGA samples was ascertained using the methylation profiles as described by Noushmehr *et al*. [6]. Briefly, level 2 methylation data was obtained from the TCGA data portal, and unsupervised hierarchical clustering was performed (Supplemental Figure 3).

### Gene signature expression in G-CIMP+ and G-CIMP- glioblastomas

Pathway activity score-heat maps were generated using the methods described above for G-CIMP+ and G-CIMP- glioblastoma samples in the CGGA and REMBRANDT data sets. Welch's two sample t-test was used to compare expression values between these two groups for each gene signature. The distribution of the *t* statistic was empirically derived by the same bootstrapping procedure as described above—1500 Monte-Carlo simulations randomizing gene identity, but not the number of over-/under-expressed genes or the data set. A two-tailed p-value was assigned, calculated as two times the percentile of the signatures’ *t* statistic in the bootstrapped distribution (or two times 1 minus the percentile, if *t* > 0.5). Signatures with p < .05 were considered significantly differently expressed between the G-CIMP+ and G-CIMP- GBMs.

In order to assess the significance of multiple signatures within the same pathway acting concordantly, a group level statistic was derived. P-values from each (non-bootstrapped) Welch's two sample t-test were combined using the Stouffer Weighted Z score. In essence, we treat the signatures as independent assays of the overall pathway's activity, and use methods derived from the meta-analysis literature to assess the evidence of pathway activity as a whole [23]. The distribution of the Z scores for each group in each data set was empirically derived by a bootstrapping procedure which performed 1500 Monte-Carlo simulations of each group. For each simulation, signatures were assigned a random gene list, keeping all other factors constant. A two-tailed p-value was assigned to the Z score from each group based on the empirical distribution of Z-scores.

### Microarray profiling of 25 independent glioblastoma specimens

All research performed was approved by IRB boards at University of California, San Diego Human Research Protections Program and were in accordance with the principles expressed at the declaration of Helsinki. Each patient was consented by a dedicated clinical research specialist prior to collection. Written consent was obtained for each patient. The consent process was approved by the ethics committee, and all records were documented in our electronic record system. The written consent from patients was also scanned into our electronic filing system. The specimens were collected at the University of California San Diego Medical Center under IRB 120345X.

In total, 25 consecutive glioblastomas were collected as fresh-frozen specimens. The specimens were secured from newly diagnosed glioblastoma patients who had not undergone temozolomide or radiation treatment. Total RNA was extracted from the specimens. Whole genome gene expression profiling was performed using Affymetrix HGU133 Plus 2.0 microarrays. Microarray data were GC Robust Multiarray Average normalized using R and the bioconductor.org package gcrma. G-CIMP status was determined using PAM as described above. The genomic data generated for this study has been made available on the Gene Expression Omnibus (http://www.ncbi.nlm.nih.gov/geo/) under accession number GSE60184.

### ERK pathway analysis by Western blotting

To determine ERK signaling pathway in 25 glioblastoma specimens, protein lysates were extracted with NP-40 buffer (1% NP-40, 20 mM Tris-HCl (pH 8.0), 137 mM NaCl, 10% glycerol,2 mM EDTA, 1 mM sodium orthovanadate, 10 μg/mL Aprotinin, 10 μg/mL Leupeptin, and 10 μg/mL Pepstatin). 50 μg of the protein lysate was fractionated by SDS-PAGE following the Western blotting using a phospho-specific anti-pERK antibody (Cell Signaling Technology, 1:1,000), anti-ERK antibody (Cell Signaling Technology, 1:2,000), anti-actin antibody (Sigma, 1:10,000), anti-Tubulin antibody (Sigma, 1:10,000), anti-Ras antibody (Cell Signaling Technology, 1: 1,000), anti-EGFR antibody (Cell Signaling Technology, 1:1,000). Band intensities were analyzed using Image J software (Bethesda, MD). The levels of pERK and ERK were quantitated based on methods previously described [24, 25]. In brief, levels of pERK and ERK were normalized to actin. Ratio of normalized pERK to ERK was then determined and compared. Statistical comparisons of the averaged scores were performed using unpaired *t*-test.

### Immunohistochemical staining of EGFR

Of the 25 microarray profiled glioblastoma specimens, 19 were present in sufficient quantity that a tissue microarray (TMA) was assembled. Regions of FFPE used to make the TMA was selected by a board-certified neuro-pathologist (H.R. and S.V.) based on the absence of necrotic tissue or normal cerebrum. Each specimen is represented by three distinct cores taken from differing region of the FFPE specimen.

The anti-EGFR monoclonal antibody (Santa Cruz Biotechnology, 1:50) was used for the IHC staining. TMA slides were incubated at 60°C for 60 min in a hybridization oven to remove secondary paraffin layers. Antigen retrieval was accomplished with incubating the sections for 10 minutes in citrate buffer at sub-boiling temperature. Primary antibodies were applied and incubated over night at 4°C. The reaction was visualized by DAB (Vector Laboratories, Burlingame, CA). The sections were counterstained with Mayer's hematoxylin and mounted with Permount™ Mounting Medium (Electron Microscopy Sciences, Hatfield, PA).

Staining of EGFR based on: 1+ (low staining), 2+ (medium staining) and 3+ (high staining) [26, 27]. Three independent cores were scored for each sample. Over 90% inter-rater reliability was observed. The discrepant scores were discussed by the three reviewers as to derive a consensus grading. The three distinct cores of each specimen were individually scored and the scores are averaged. Statistical comparisons of the averaged score were performed using the non-parametric Mann-Whitney test with Graphpad Prism (GraphPad Software, Inc.).

### Cell culture, plasmid constructs, and transfection

Human glioma cells U87MG are purchased from American Type Culture Collection (Manassas, VA). Murine *Ink4a-Arf−/−* cells were kindly provided by Dr. Oren Becher (Duke University Medical Center). The cells were propagated at 37°C (humidified atmosphere containing 5% CO2) in Dulbecco's modified Eagle medium supplemented with 10% fetal calf serum, 2 mM L-glutamine, 100 U/mL penicillin G sodium, and 100 mg/mL streptomycin sulfate (Gibco). For 2HG treatment, U87 cells were treated with 2HG (1mM, Sigma) for >10 passages.

The wild-type human *IDH1* and *IDH1-R132H* mutant (c.395G>A) were generously provided by Dr. Kun-Liang Guan(University of California, San Diego) and Yue Xiong (Fudan University, China). The constructs were confirmed by Sanger sequencing. The wild-type EGFR, EGFRvIII, and H-Ras (G12V) constructs were generously provided by Dr. Frank Furnari (University of California, San Diego). Retrovirus packaging and infection were performed as previously described [28] and stably infected cells were generated by selection with puromycin (1 μg/ml) for 5 days, G418 (600 μg/ml) for 2 weeks, or Hygromycin (100 μg/ml) for 2 weeks prior to the subsequent experiments. Gefitinib was purchased from SelleckChem and used at 10 μM.

### DNA extraction, PCR Amplification, Purification, and Direct DNA sequencing

Genomic DNA was extracted from FFPE sections using QIAamp DNA FFPE Tissue Kit (Qiagen) according to the manufacturer's instructions. The DNA concentration was determined with Nanodrop (Thermo Scientific). Exon 4 of the *IDH1* gene was amplified with PCR as previously described [29] using the following primers: Forward, 5' CGGTCTTCAGAGA-AGCCATT 3', and Reverse 5' GCAAAATCA-CATTATTGCCAAC 3'. The products were purified using QIAquick PCR Purificaiton Kit (Qiagen) and all purified PCR amplicons of *IDH1* were subjected gel electrophoresis (2% agarose), followed by QIAquick PCR Purification (Qiagen) and Sanger sequencing for detection of specific mutations.

### Chromatin Immunoprecipation (ChIP) assay

ChIP assays were performed as described before [30]. In brief, after crosslinking with formaldehyde, cells were lysed and chromatin was harvested and fragmented by micrococcal nuclease digestion (5,000U/sample for 20 min). The chromatin was then subjected to the immunoprecipitation using H3K9me3 (Active Motif) and H3K27me3 (Abcam) antibodies followed by DNA purification. Histone H3 antibody was used as positive control. Primers sequences used for amplifying ChIP products are: EGFR, Forward: 5' GGACACTTAGCCTCTCTAAA 3', and Reverse: 5' GGGAAACTGCTCCTTTATTC 3'; H-Ras, Forward: 5' CAGATTGAAGGATGCCTAGA 3', and Reverse: 5' GCATCTCCTAATCTCCTCTG 3'. Normalized Ct (ΔCt) values were calculated by substracting the Ct obtained with input DNA from that obtained with immunoprecipitated DNA (ΔCt=Ct (IP)-Ct (Input)). Relative fold enrichment of H3K9me3 or H3K27me3 at the target site was then calculated using percent of positive control. Changes related to expression of wild type *IDH1* or *IDH1- R132H* mutant was then represented by fold change relative to cells infected with empty vector.

### SUPPLEMENTARY MATERIAL FIGURES AND TABLE



### SUPPLEMENTARY MATERIAL AND TABLE


