# Supplementary material for: Epigenetic suppression of EGFR signaling in G-CIMP+ glioblastomas
Source: Oncotarget. 2014 Aug 16;5(17):7342–56. doi: 10.18632/oncotarget.2350 (PMC4202127; doi:10.18632/oncotarget.2350)
Supplement: Supplementary file 1 [file oncotarget-05-7342-s001.pdf]

Epigenetic suppression of EGFR signaling in G-CIMP+ glioblastomas

Supplementary Material

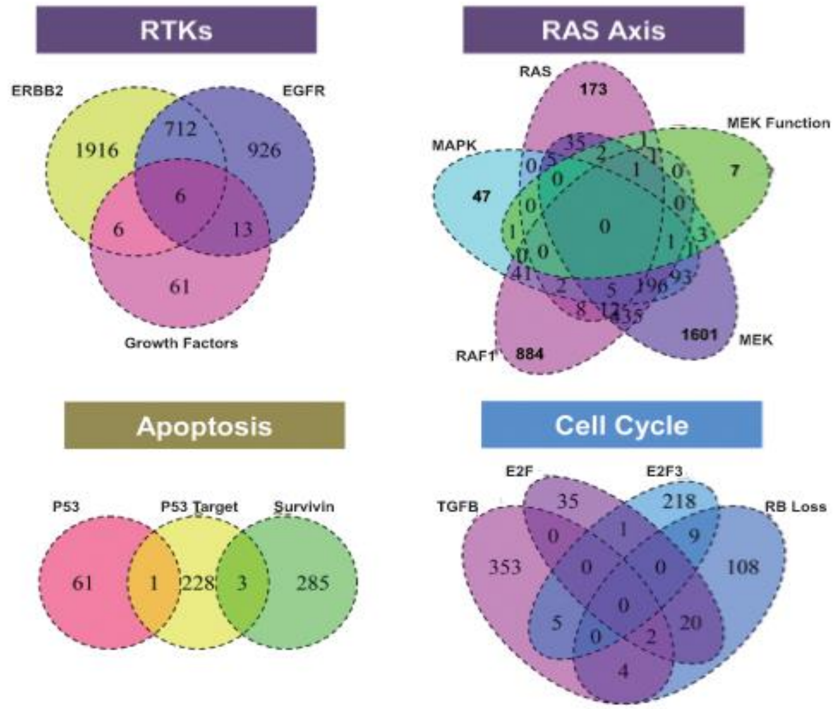

Supplemental Figure 1: Venn diagram of the overlap in signature gene list.

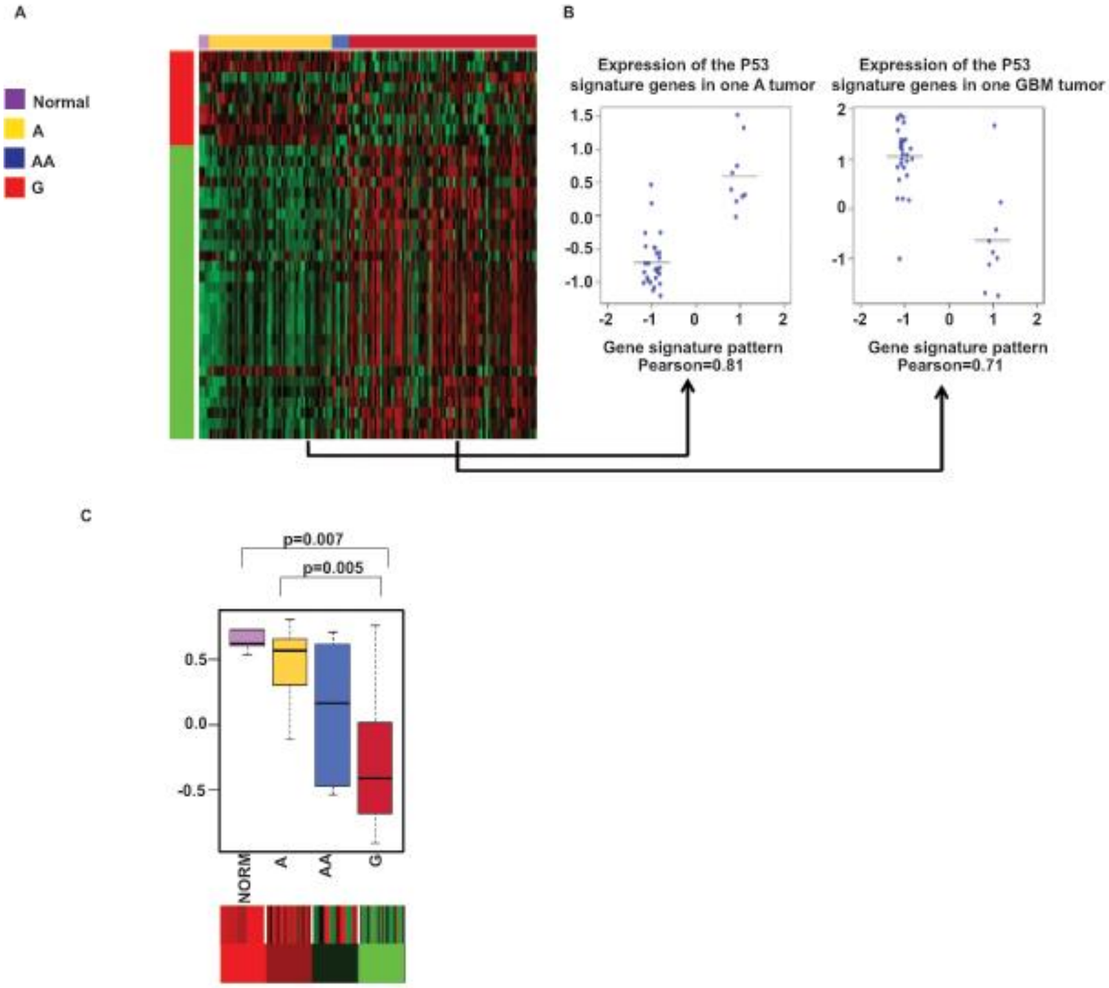

Supplemental Figure 2: Schema depicting the derivation of the pathway activity score. (A) Heat map of the expression pattern of the p53 gene signature in the CGGA. Lavender = normal brain, yellow = grade II astrocytoma (A), blue = grade III astrocytoma (AA), red = glioblastoma (G). (B) Activity score is defined as the following manner: Each gene in the signature that was over-expressed was assigned the value of +1; genes that were under-expressed were assigned -1. The normalized expression values of the signature genes in the clinical specimen are then plotted against these assigned values, and Pearson Correlation Coefficient was calculated as the pathway activity. The correlation ranged from -1 (denting low pathway activity) to +1 (denoting high pathway activity). (C) The pathway activity values (top) were averaged and displayed as a heat map (bottom).

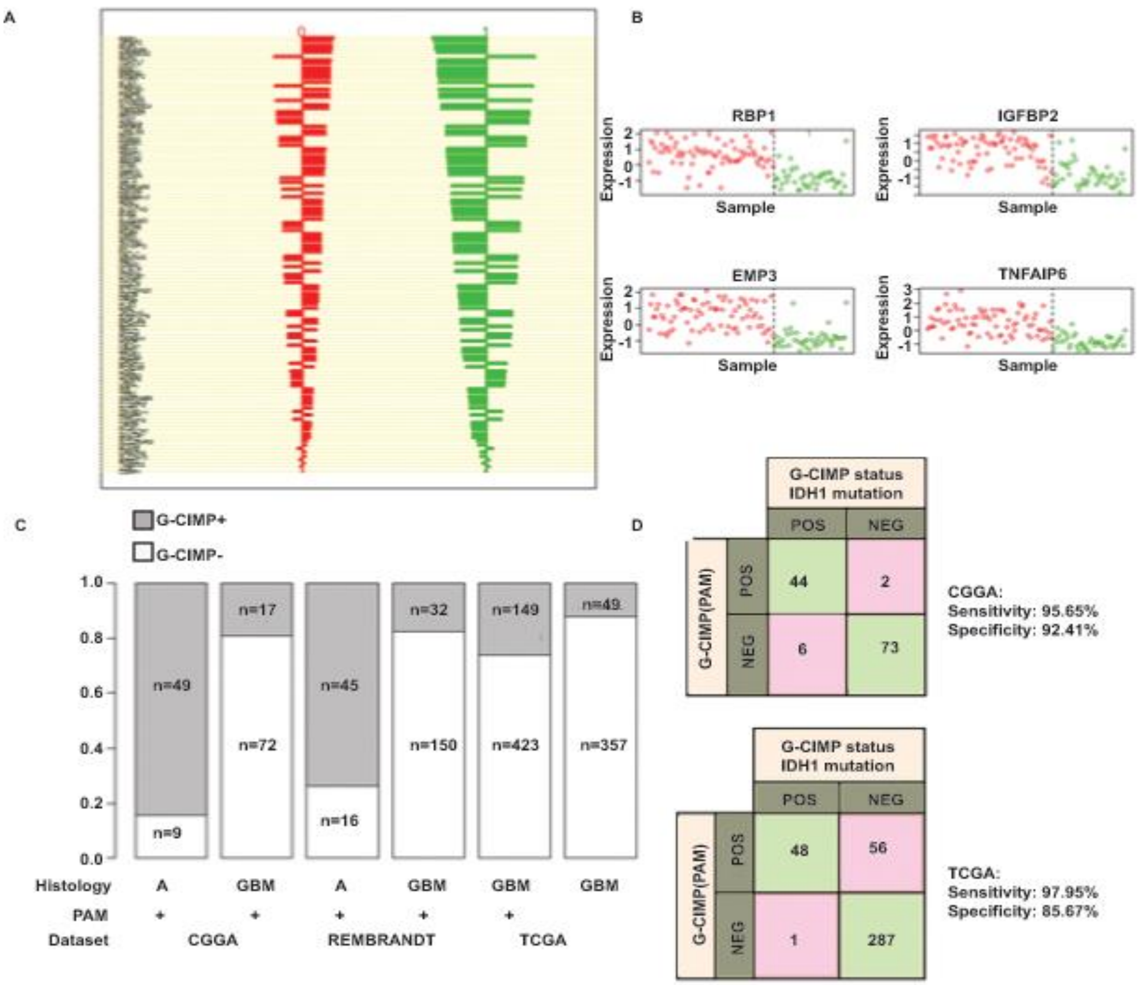

Supplemental Figure 3: PAM classification of G-CIMP status. (A) Plot of the centroids for the G-CIMP classifier genes [1] using the CGGA dataset. (B) Expression level of the top four classifier genes and determination of threshold for G-CIMP status. (C) Proportion of glioblastomas classified as G-CIMP+ and G-CIMP- based on the mRNA classifiers. (D) Sensitivity and specificity of G-CIMP status determination by PAM. In the CGGA, IDH1 mutation was used as the gold standard for defining G-CIMP+ status [7]. In the TCGA, hierarchical clustering of methylation was used as the gold standard for G-CIMP+ status [22]. The PPV and NPV for the CGCG dataset was 88 and 97.33% respectively. The PPV and NPV for the TCGA dataset was 97.95 and 83.67%, respectively.

Table S1

	#NORM	#A	#AA	#GBM	#Unique Human Genes
CGGA	5	58	8	89	19416
REMBRANDT	28	61	45	182	18599
TCGA	10	n/a	n/a	406	17814

Supplemental References

1. Loboda A, Nebozhyn M, Cheng C, Vessey R, Huang P, Dai H and Watters JW. Biomarker discovery: identification of a growth factor gene signature. Clin Pharmacol Ther. 2009; 86(1):92-96.
2. Bild AH, Potti A and Nevins JR. Linking oncogenic pathways with therapeutic opportunities. Nat Rev Cancer. 2006; 6(9):735-741.
3. Dry JR, Pavey S, Pratilas CA, Harbron C, Runswick S, Hodgson D, Chresta C, McCormack R, Byrne N, Cockerill M, Graham A, Beran G, Cassidy A, Haggerty C, Brown H, Ellison G, et al. Transcriptional pathway signatures predict MEK addiction and response to selumetinib (AZD6244). Cancer Res. 2010; 70(6):2264-2273.
4. Coutant C, Rouzier R, Qi Y, Lehmann-Che J, Bianchini G, Iwamoto T, Hortobagyi GN, Symmans WF, Uzan S, Andre F, de The H and Pusztai L. Distinct p53 gene signatures are needed to predict prognosis and response to chemotherapy in ER-positive and ER-negative breast cancers. Clin Cancer Res. 2011; 17(8):2591-2601.
5. Mirza A, Wu Q, Wang L, McClanahan T, Bishop WR, Gheyas F, Ding W, Hutchins B, Hockenberry T, Kirschmeier P, Greene JR and Liu S. Global transcriptional program of p53 target genes during the process of apoptosis and cell cycle progression. Oncogene. 2003; 22(23):3645-3654.
6. Salz W, Eisenberg D, Plescia J, Garlick DS, Weiss RM, Wu XR, Sun TT and Altieri DC. A survivin gene signature predicts aggressive tumor behavior. Cancer Res. 2005; 65(9):3531-3534.
7. Tian M and Schiemann WP. The TGF-beta paradox in human cancer: an update. Future Oncol. 2009; 5(2):259-271.
8. Ertel A, Dean JL, Rui H, Liu C, Witkiewicz AK, Knudsen KE and Knudsen ES. RB-pathway disruption in breast cancer: differential association with disease subtypes, disease-specific prognosis and therapeutic response. Cell Cycle. 2010; 9(20):4153-4163.
9. Locatelli G, Bosotti R, Ciomei M, Brasca MG, Calogero R, Mercurio C, Fiorentini F, Bertolotti M, Scacheri E, Scaburri A, Galvani A, Pesenti E, De Baere T, Soria JC, Lazar V and Isacchi A. Transcriptional analysis of an E2F gene signature as a biomarker of activity of the cyclin-dependent kinase inhibitor PHA-793887 in tumor and skin biopsies from a phase I clinical study. Mol Cancer Ther. 2010; 9(5):1265-1273.
